# Understanding Peri-Implantitis as a Plaque-Associated and Site-Specific Entity: On the Local Predisposing Factors

**DOI:** 10.3390/jcm8020279

**Published:** 2019-02-25

**Authors:** Alberto Monje, Angel Insua, Hom-Lay Wang

**Affiliations:** 1 Department of Periodontology, Universitat Internacional de Catalunya, 08195 Barcelona, Spain; 2 Division of Periodontics, CICOM Periodoncia, 06011 Badajoz, Badajoz, Spain Santiago de Compostela, Spain; ainsua@umich.edu; 3 Department of Periodontics and Oral Medicine, University of Michigan School of Dentistry, Ann Arbor, MI 48109, USA; homlay@umich.edu

**Keywords:** peri-implantitis, peri-implant endosseous healing, dental implantation, dental implant, alveolar bone loss

## Abstract

The prevalence of implant biological complications has grown enormously over the last decade, in concordance with the impact of biofilm and its byproducts upon disease development. Deleterious habits and systemic conditions have been regarded as risk factors for peri-implantitis. However, little is known about the influence of local confounders upon the onset and progression of the disease. The present narrative review therefore describes the emerging local predisposing factors that place dental implants/patients at risk of developing peri-implantitis. A review is also made of the triggering factors capable of inducing peri-implantitis and of the accelerating factors capable of interfering with the progression of the disease.

## 1. Introduction

Implant dentistry, as a scientific discipline, has grown rapidly over the last four decades with the aim of facilitating early and effective osseointegration affording successful long-term outcomes. Over these years, the onset of complications has been neglected as representing only isolated events. Nowadays, however, due to the increase in prevalence of such problems, one of the major endeavors in this field is the prevention and efficient management of biological complications referred to as peri-implant diseases [1,2].

According to the bacterial theory, peri-implantitis by definition is a chronic inflammatory condition associated with a microbial challenge [3]. Nevertheless, in some cases there may be immunological reasons behind marginal bone loss [4,5,6] not primarily related to biofilm-mediated infectious processes [7]. Accordingly, a change from a stable immune system, seen during maintained osteointegration, to an active system may lead to the rejection of foreign bodies [7]. In this regard, implant surfaces types, surface wear, or contaminated particles may enhance these immunological reactions [8].

The conversion process from peri-implant mucositis mirrors the progression from gingivitis to periodontitis, with the constant formation of plaque features in the peri-implant tissues, characterized by erythema, bleeding, exudation, and tumefaction. At histological level, the establishment of B- and T-cell-dominated inflammatory cell infiltrates has been evidenced [9]. However, the clinical and histopathological characteristics during the conversion process are still not fully clear. Following conversion, peri-implantitis progresses in a nonlinear and accelerated manner [10].

The epidemiology of peri-implantitis varies widely depending on the given case definition. There has been important controversy regarding the threshold defining physiological peri-implant bone loss. As such, unspecific ranges have been observed in meta-analyses with heterogeneous case definitions. In 2012, the VIII European Workshop of Periodontology underscored that the diagnosis of peri-implantitis should be given on a longitudinal basis of overt progressive bone loss with clinical signs of inflammation [11]. In this regard, a threshold of ≥2 mm of peri-implant bone loss could be accepted for the diagnosis of peri-implantitis. More recently, the American Academy of Periodontology and the European Federation of Periodontology have jointly proposed a case definition based on a threshold of ≥3 mm [12]. Recent meta-analytical data have suggested the prevalence of peri-implantitis to be 18.5% at patient level and 12.8% at implant level [13], though the prevalence at patient level ranges widely between 1 and 47% [14]. Regardless of the diagnostic criteria proposed, peri-implantitis has been shown to be a site-specific condition. In contrast to periodontitis, which manifests with generalized loss of support, peri-implantitis commonly progresses conditioned by factors predisposing to biofilm accumulation which, under susceptible conditions, triggers a complex inflammatory response.

Strong evidence suggesting an increased risk of peri-implantitis has been obtained in subjects with poor personal- and professional-administered oral hygiene measures, and in individuals with a history of periodontitis [15,16]. Even though other factors and deleterious habits such as smoking [17] or hyperglycemia [18] have been identified as potential risk factors, there is a need for further and stronger evidence to validate their influence upon the development of peri-implantitis [3].

Moreover, in a site-specific condition, attention should focus on those factors which locally might be predisposing for the onset and progression of the disease [19]. Accordingly, the 2017 World Workshop identified evidence linking peri-implantitis to factors that complicate access to adequate oral hygiene, that is, those local conditions that predispose certain implants to develop disease [12].

## 2. Significance of Terminology for Reaching Consensus

As mentioned above, peri-implantitis and periodontitis occur more frequently under certain systemic conditions and in the presence of deleterious habits. For instance, it is known that major periodontal disease risk factors such as smoking and diabetes alter the epigenetics by downregulating the genic expression of bone matrix proteins that could influence the pathway from peri-implant mucositis to peri-implantitis by suppressing specific transcription factors for osteogenesis, or by activating certain transcription factors for osteoclastogenesis [20,21]. Hence, these systemic conditions may increase the risk of suffering peri-implant diseases.

On the other hand, emerging data point to the influence which certain local factors might have upon the onset and development of disease, since they induce plaque accumulation. These are the so-called predisposing factors. Terminologically, a predisposing factor is a condition that places the given element (dental implant)/individual (patient) at risk of developing a problem (peri-implantitis). In this regard, it is also of interest to underscore that a triggering factor, if not controlled after diagnosing and arresting (or not arresting) the problem (peri-implantitis), represents a perpetuating element that maintains the problem after it has become established [22]. Accelerating factors are therefore defined as those conditions that do not play a role in the onset of a problem (peri-implantitis) but can influence its progression.

## 3. Are Dental Implants Predisposed to Develop Biological Complications?

The evolution of dental implants and their incorporation to routine practice to restore function and aesthetics of lost or failing dentition have been described as one of the most revolutionary and innovative developments of the twentieth century. In fact, early dental implants were developed with a minimally rough surface microdesign. At that stage in modern implant dentistry, the osseointegration process proved slower and less effective. Long-term findings reported that these implants moreover tended to fail more frequently in the maxilla compared with the mandible. In addition, mean marginal bone loss using primitive implant–abutment connections was shown to be 1.5 mm, with an annual progressive bone loss of 0.1 mm [23,24].

With the development of new technology, the vast majority of commercial implants now have modified (moderately rough) surfaces with the primary aim of securing earlier osseointegration [25]. The incorporation of more biologically acceptable connections may be able to restrict inflammatory infiltration and thus minimize physiological bone loss. Indeed, a clinical study showed that 96% of the implants with a marginal bone loss of >2 mm at 18 months had lost 0.44 mm or more at 6 months postloading [26]. Thus, early healing dictates the long-term life of dental implants and the occurrence of biological complications, as it can be assumed that the establishment of a more anaerobic environment results in greater susceptibility to progressive bone loss.

Advances in the knowledge of bone biology and translational medicine summed to the development of novel armamentaria allow the clinician to minimize physiological bone remodeling. In this regard, excessive physiological bone remodeling (loss) may create a niche for the harboring of periopathogenic microorganisms that can lead to the development of implant biological complications.

## 4. Peri-Implant Monitoring: Diagnostic Accuracy of Clinical Peri-Implant Parameters

The prompt diagnosis of peri-implant disease is crucial to achieve favorable therapeutic outcomes. While the nonsurgical treatment of peri-implant mucositis is effective, the management of peri-implantitis proves more challenging [27]. Along these lines, it is worth mentioning that the severity and extensiveness of the lesion are crucial factors for successful and maintainable outcomes.

**Figure 1:**
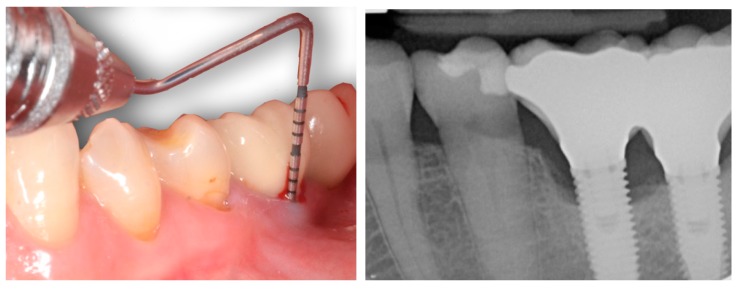
Bleeding on probing and increased probing pocket depth are clinical signs of peri-implantitis. The final diagnosis should be based on the correlation of the clinical data to the radiographic findings.

**Figure 2:**
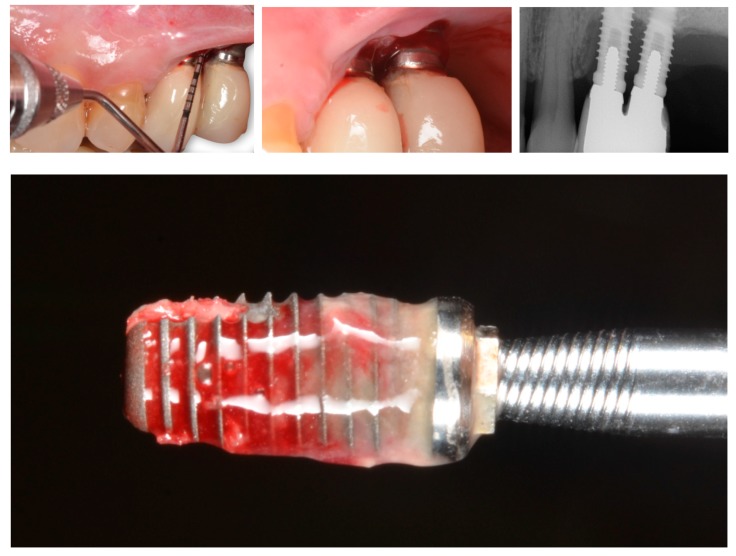
Bleeding on probing and increased probing pocket depth are clinical signs of peri-implantitis. The final diagnosis should be based on the correlation of the clinical data to the radiographic findings. When bone loss is advanced, implant removal is often the most predictable option for dealing with peri-implantitis.

Peri-implantitis develops with progressive bone loss and signs of inflammation. As such, in order to secure an accurate diagnosis, the classical signs of inflammation (i.e., warmth, reddening, tumefaction) and an increased probing depth compared to baseline (assuming a measurement error) must be present [12] (Figure 1; Figure 2), as evidenced by clinical (Table 1) and preclinical studies. Interestingly, during the progression of ligature-induced experimental peri-implantitis, all the clinical parameters are worse due to the degree of inflammation present [28,29,30,31].

In this sense, it should be mentioned that disagreement persists concerning the sensitivity of bleeding on probing (BOP) and suppuration as diagnostic criteria. For instance, a human study showed the probability of positive BOP at a peri-implant site with a probing depth of 4 mm to be 27% [32]. The odds for positive BOP was seen to increase 1.6-fold per 1 mm of further probing depth. It has been further evidenced that BOP might be influenced by patient-related factors such as smoking [32]. In fact, understanding of the morphological differences of the periodontal apparatus compared with the peri-implant tissues supports the possibility that the former responds differently to mechanical stimulation. This might explain the poorer sensitivity in the detection of peri-implant diseases compared with periodontal diseases. Likewise, suppuration has been reported in about 10–20% of all peri-implant sites [28,33,34,35,36]. Hence, suppuration does not seem to exhibit high sensitivity in the diagnosis of peri-implantitis.

In sum, clinical monitoring of peri-implantitis using a periodontal probe is indicated at each maintenance visit, with the purpose of preventing major biological complications. Nevertheless, the definite diagnosis should be based on the radiographic findings compared to baseline.

## 5. Local Predisposing Factors

### 5.1. Significance of Soft Tissue Characteristics

The characteristics of the periodontal soft tissues and their association to periodontal conditions have been the subject of debate [41,42,43,44,45]. Based on the existing literature, it seems that attached keratinized gingiva is beneficial in patients with deficient oral hygiene. In contrast, patients with adequate personal- and professional-administered oral hygiene measures do not benefit from attached keratinized gingiva. In fact, movable mucosa facilitates the penetration of biofilm into the crevice, which would trigger the activation of neutrophils and lymphocytes [43]. Hence, in patients not adhering to adequate hygiene, the presence of keratinized attached gingiva might play a pivotal role in the prevention of the disease, in particular in the presence of subgingival restorations.

**Figure 3:**
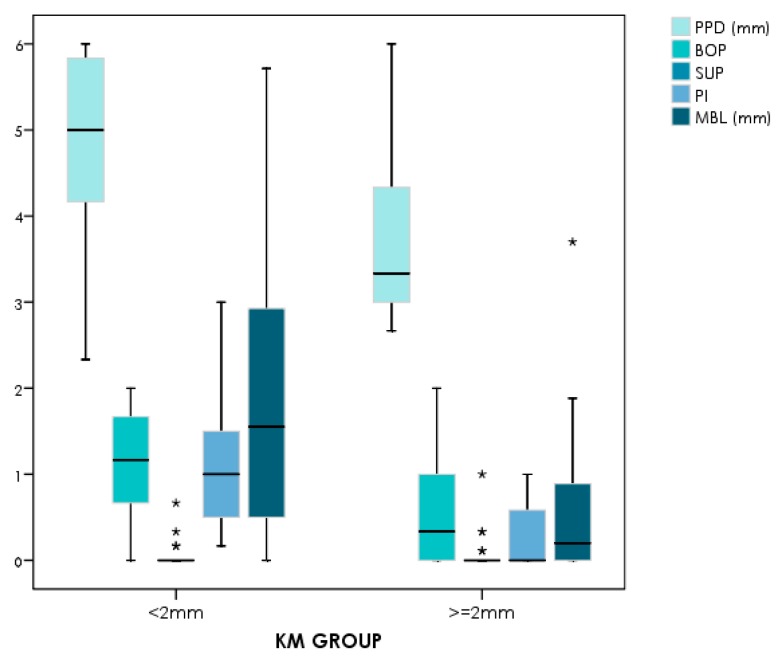
Comparative plot showing the clinical and radiographic differences between <2 mm versus ≥2 mm of peri-implant keratinized mucosa in erratic maintenance compliers [49]. Note: * stand for the outliers

**Figure 4:**
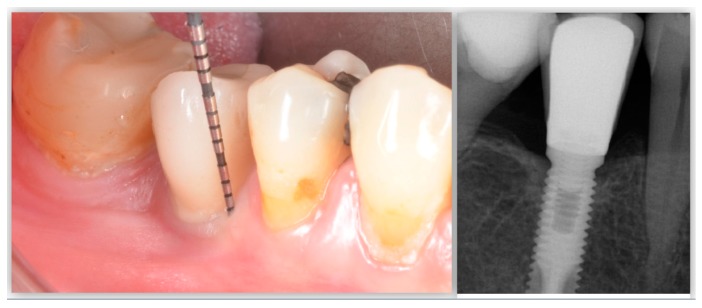
Representative case of an erratic maintenance complier with inadequate personal-administered oral hygiene presenting with healthy clinical and radiographic peri-implant conditions in the presence of 2 mm of keratinized and attached mucosa.

The influence of keratinized mucosa around dental implants has not been without controversy (Table 2). Early findings indicated that a lack of keratinized mucosa was not associated with less favorable peri-implant conditions [46]. More recent data have shown a wide band of keratinized mucosa to favor improved scores referred to as plaque index, modified gingival index, mucosal recession, and attachment loss [47]. Likewise, it has been demonstrated that the presence of keratinized mucosa around dental implants has a positive impact upon the immunological features, with a negative correlation to prostaglandin E2 levels [48]. This is due in part to a reduced inflammatory condition as a consequence of less discomfort during personal-administered oral hygiene. In fact, two recent clinical studies have shown the presence of ≥2 mm of keratinized mucosa to be crucial for the prevention of peri-implant diseases in erratic maintenance compliers [49] (Figure 3; Figure 4).

Thus, a lack of keratinized mucosa in patients with inadequate oral hygiene could be regarded as a predisposing factor for peri-implant diseases, since it is associated with more recession, less vestibular depth, and more plaque accumulation, which, in turn, may be predisposing to inflammation (i.e., peri-implantitis).

### 5.2. Surgical Predisposing Factors

#### 5.2.1. Significance of Implant Malpositioning as an Iatrogenic Factor: Critical Bone Dimensions

In the 2017 World Workshop on the classification of Periodontal and Peri-Implant Diseases and Conditions, implant malpositioning was suggested to be a predisposing factor for peri-implantitis due to the limited access for adequate oral hygiene often associated with these implant-supported restorations. If fact, a retrospective study associated implant malpositioning (OR = 48), occlusal overload (OR = 18.7), prosthetic problems (OR = 3.7), and bone grafting procedures (OR = 2.4) with peri-implantitis [54]. An early survey of cases reported in the literature as corresponding to peri-implantitis, following evaluation by a group of independent experts in the field, agreed that >40% of the implants diagnosed with peri-implantitis presented with a too-buccal position, with perfect interexaminer agreement (k = 0.81) [55]. This is in contrast to a four-year clinical study which found implants with residual buccal dehiscence defects to be more prone to develop peri-implantitis [56].

**Figure 5:**
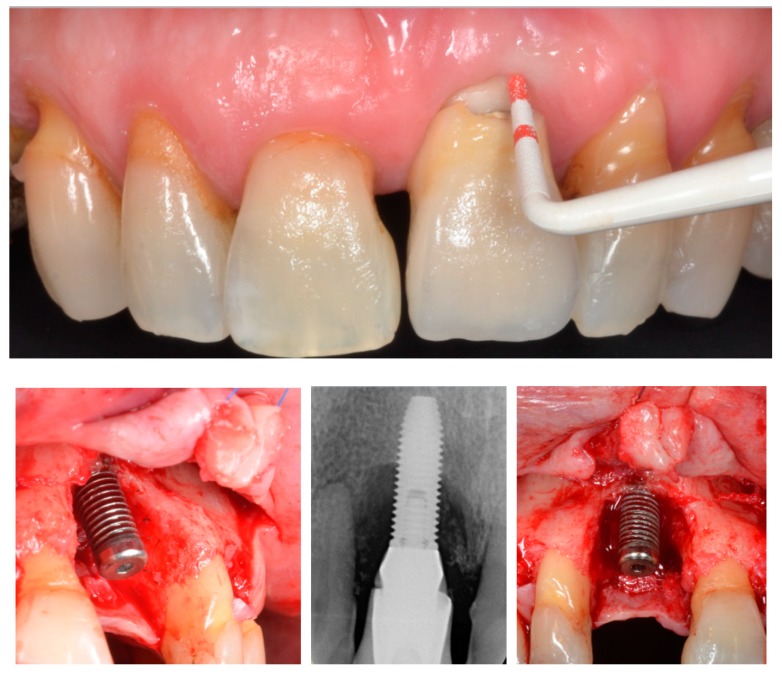
A critical buccal bone thickness of 1.5 mm is essential for preventing excessive physiological and pathological bone loss as a consequence of early avascular necrosis leading to peri-implant bone loss and thus to an increased risk of surface contamination.

**Figure 6:**
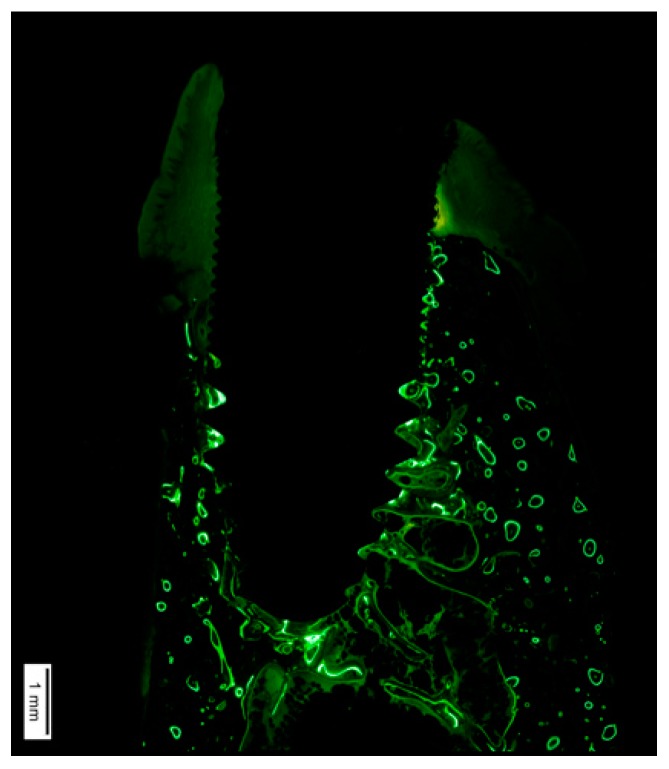
Histological analysis with fluorescent dyes illustrating excessive bone loss as a consequence of ligature-induced peri-implantitis. Note that the lack of fluorescence on the buccal side demonstrates the severe vertical bone resorption that occurs after physiological bone remodeling due to the insufficient critical buccal bone thickness (<1.5 mm).

A comprehensive understanding of bone biology is crucial to conceive implant positioning, in particular, too-buccal positioning, as a predisposing factor for peri-implantitis. In a healed ridge, the alveolar process is composed of cortical bone at the outer side, while cancellous bone is featured in the inner structure. The cortical bone receives a blood supply branched from the outside through blood vessels of the periosteal surface, and from the inside from the endosteum [57]. When an implant is inserted with an open-flap procedure, elevation of the periosteum eliminates the periosteal blood supply from the outside. The same process occurs from the inside, since insertion of the implant interrupts the endosteal blood supply. This phenomenon of avascular necrosis is well known in bone biology [58] (Figure 5). A recent study has demonstrated that the critical buccal bone thickness for preventing marked physiological buccal–lingual bone resorption is 1.5 mm. In the absence of this thickness, more pronounced peri-implantitis may occur as a consequence of the microrough surface exposed to the oral cavity-facilitating surface contamination and the chronification of peri-implant infection [59] (Figure 6).

**Figure 7:**
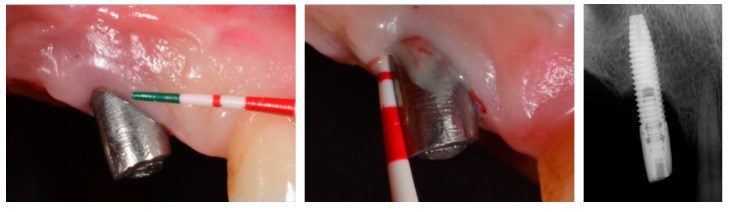
Inadequate apico-coronal implant positioning may favor the establishment of a microbial anaerobic environment that can be predisposing to progressive pathological peri-implant bone loss.

Likewise, apico-coronal implant positioning might dictate the long-term stability of the peri-implant tissues (Figure 7). Based on the hypothesis that too-apical implant positioning may favor the establishment of a microbial anaerobic environment, it is advised that implants be placed within the apico-coronal safety threshold. A recent retrospective analysis has validated this idea. Kumar et al., in nonsplinted single implants in function for at least five years, demonstrated that implant placement at a depth of ≥6 mm from the cementoenamel junction of the adjacent teeth is more commonly associated with peri-implantitis (OR = 8.5) [60]. Similarly, it should be noted that other factors could increase bone loss in these scenarios such as the type of implant–abutment connection (external vs internal vs conical) [61], number of abutment connection/disconnection [62], or the increased difficulty in removing cement remnants in case of cemented restorations [63].

**Figure 8:**
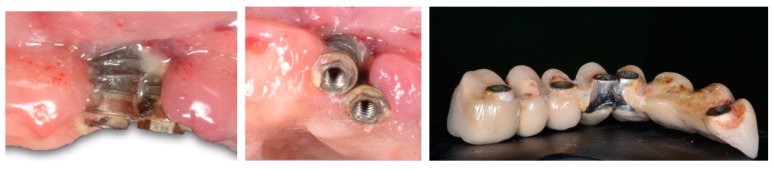
Incorrect implant positioning predisposes dental implants to peri-implant diseases due to the inability to perform correct oral hygiene.

The mesiodistal implant position could be regarded as a predisposing factor for peri-implant bone loss, leading to peri-implantitis due to two main factors: (1) inadequate access for performing correct oral hygiene; and (2) excessive physiological bone remodeling if no safety distance is ensured between two adjacent dental implants or one implant with the adjoining dentition (Figure 8). Classically, the recommendation was to leave 3 mm between dental implants [64]. Even though this is no longer applicable to current implant dentistry due to advances in implant–abutment designs, a safety distance must be observed in order to avoid avascular necrosis of the interimplant cortical bone, with sufficient space to favor adequate personal oral hygiene.

#### 5.2.2. Implant Insertion Torque and Its Interplay with the Hard Tissue Substratum

**Figure 9:**
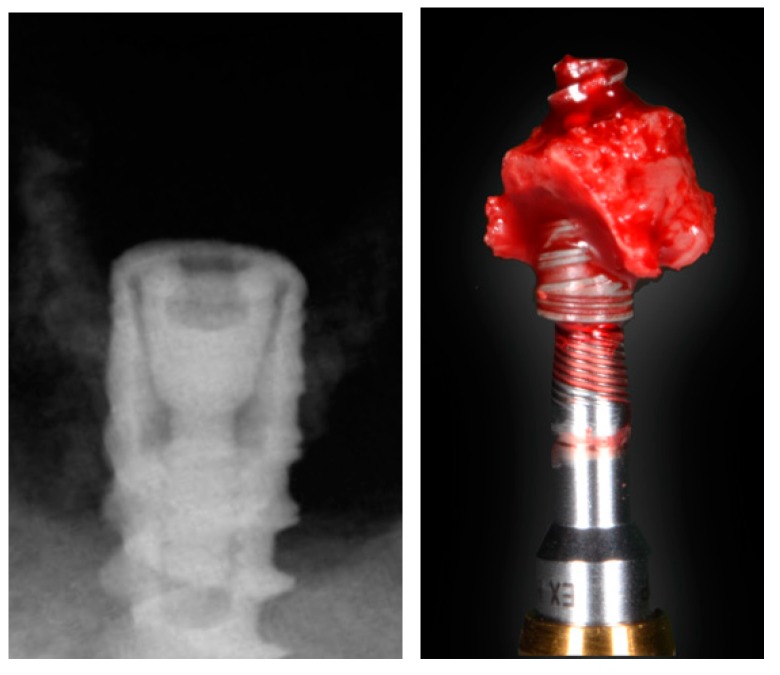
Implant removed four months after placement in the mandible. Note that the implant macrodesign, together with a highly corticalized bone structure, have induced excessive bone loss extending on the coronal portion of the implant and creating bone necrosis on the apical part. The severe bone resorption in the coronal area might have been predisposing to peri-implantitis as a biological complication if the implant had not been removed.

Implant placement in low-density bone can prove challenging. Thus, in order to ensure adequate primary stability and reduce early osseointegration implant failures, adaptation of the drilling protocol to the bone features has been recommended [65]. In fact, modifications in implant macrodesign, the use of osteotome condenser drills, and underpreparation of the implant socket may increase primary stability and osseointegration [65]. It is important to note that the connections between the trabecular mesh give cancellous bone the capacity to bear loads [66]; atraumatic surgical procedures therefore minimize the risk of bone loss. Thus, the use of drills to condense and densify trabecular bone might disrupt the connectivity of the trabecular network, reduce the capacity of bone to transmit occlusal forces, and result in weak bone that might not guarantee secondary stability due to higher bone turnover [66]. In fact, excessive compression of peri-implant bone by using osteotomes or increased torque may lead to 22–50% more crestal bone loss than conventional implantation [67,68] and also to a 41% reduction in the amount of bone-to-implant contact [69]. Such mechanical devices may damage the canalicular network of the trabecular bone, leading to a change in fluid flow mechanisms, impairment of mechanical stimulation, and delayed new bone formation [69]. Similar undesirable effects may be caused by excessive torque [70], leading to bone compression and delaying bone healing [71] (Figure 9). Areas with minimal bone-to-implant contact and therefore low strain seem to promote faster osteoblast differentiation [66,71,72]. During the first weeks, bone in contact areas around the implant threading is reabsorbed, and bone formation occurs earlier in contact-free areas [73].

The assessment of bone architecture is also relevant for implant drilling [74]. Larger osteocyte necrosis areas were found in trabecular bone versus cortical bone (550 versus 1400 µm, respectively) [65]. A similar increase in osteocyte damaged area was found when drilling speed was raised from 500 to 1500 rpm (600 versus 1400 µm, respectively) [65]. When using a 1.6 mm drill, a distance of 1050 µm of bone damage from the osteotomy center is expected, whereas the distance is about 1400 µm if a 5 mm drill is used [74]. The larger the drill diameter, the greater the tangential speed and centrifugal force, and therefore also the drilling power and energy transmitted to the bone. Lower values of early bone area formation around 5 mm implants versus 3.75 mm implants were found, and the use of large-diameter drills may be one of the underlying reasons [75]. Recently, simplified protocols have been proposed to reduce drilling time. Some authors reported no detrimental effects upon bone formation [75], but less bone formation was found in early stages in other studies [76]. It is important to note that simplified protocols might increase bone compression [76]. Moreover, the drill torque energy applied to the bone increases as the diameters of two consecutive drills increase. This fact might elevate the bone temperature and consequently the area of bone damage [65]. Further, other approaches, such as ultrasonic site preparation, have evidenced better preservation of the bone microarchitecture, resulting in a faster healing response [77].

### 5.3. Significance of Prosthetic Design

**Figure 10:**
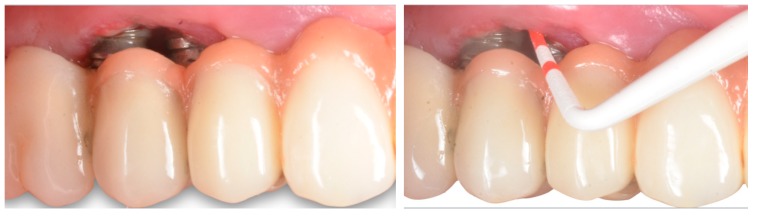
A hybrid prosthesis does not facilitate adequate oral hygiene and favors plaque retention, thereby triggering peri-implantitis.

Assuming the role of biofilm and its bacterial byproducts in the onset and progression of peri-implantitis, it is conceivable that retentive prosthetic components may promote inflammation (Figure 10). In this regard, Serino and Strom demonstrated that regardless of adequate oral hygiene of the natural dentition in partially edentulous patients, prosthetic design plays a major role in plaque accumulation around implant-supported prostheses. The authors found that adequate oral hygiene could not be performed in 53 out of 58 implants, and that peri-implantitis therefore could be attributed to deficient access for personal oral hygiene [78]. This is a typical scenario in hybrid prostheses, where esthetic requirements are satisfied but long-term implant maintenance is jeopardized owing to poor access for oral hygiene. Similarly, bone-level, implant-supported single crowns with an emergence angle of over 30 degrees and a convex profile have been shown to be factors strongly associated with peri-implantitis. This was not consistent with the findings in tissue-level implants [79]. Hence, convexities and marked emergence profiles should be avoided in the design of single crowns. In any case, patients should be comprehensively instructed to use interproximal brushes to remove food debris or plaque within the implant surroundings [2].

Into the bargain, conceiving that excessive early bone resorption is often associated with greater late bone loss [26], prosthetic factors associated with minimal physiological bone loss should be noted. In this sense, longer transmucosal abutments (>2 mm) [80] and internal connection (including platform-switching [81] and Morse cone connections [82]) have demonstrated efficient preservation of the peri-implant hard tissue levels.

## 6. Local Precipitant Factors

The literature describes a few factors (so-called precipitant factors) associated with the triggering of inflammation within the peri-implant sulcus.

### 6.1. Residual Submucosal Cement

While screw-retained restorations do not necessarily outperform cement-retained prostheses, the presence of residual cement has been shown to have a deleterious effect upon the peri-implant tissues. Wilson et al. demonstrated the triggering role of residual cement, since 81% of the cases developed peri-implantitis, with spontaneous resolution in 74% following mechanical removal of the excess cement [63]. Likewise, Linkevicius et al. demonstrated the effect of residual cement upon peri-implant tissue response. In this scenario, 85% of the cases developed peri-implant disease [83]. Similar findings were obtained by Korsch et al. in a later study affording further insight into the effect of cement type upon the development of pathological complications. It was seen that while methacrylate cement was present in 62% of the suprastructures, zinc oxide eugenol cement could not be detected [84]. As a matter of fact, it was seen that the clinical and radiographic peri-implant conditions were generally unfavorable for implant-retained suprastructures using methacrylate cement, irrespective of cement excess [84]. In view of the significance of the presence of residual cement upon peri-implant tissue stability, it is advisable to use radiopaque cement if needed, with the aim of promptly detecting and removing it.

### 6.2. Residual Dental Floss

The remnants of floss in the peri-implant sulcus have also been regarded as a triggering factor for peri-implantitis. Van Velzen et al. reported 10 cases with progressive peri-implantitis related to floss remnants. Interestingly, in 90% of the cases, the inflammation resolved spontaneously after mechanical removal of the floss remnants [85]. Thus, caution is required when providing personal oral hygiene instructions involving the use of floss, and patients should be further encouraged to employ interproximal brushes.

## 7. Local Accelerating Factors: Influence of Surface Topography upon Progressive Bone Loss

**Figure 11:**
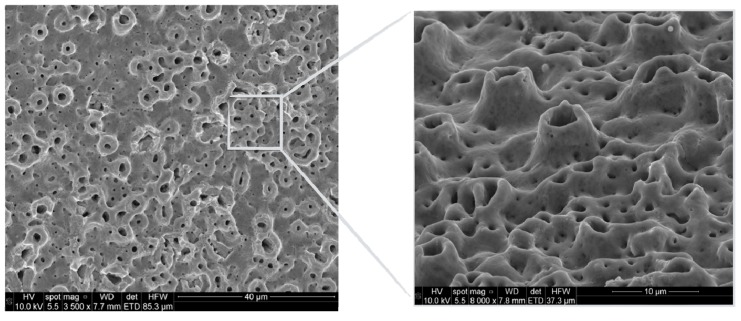
Moderately rough topographic characteristics (RA~1.3 µm) may induce chronification of the inflammatory condition by harboring pathogenic bacteria. This, in turn, may influence the therapeutic outcome. Note the scanning electron microscopic features of the implant surface under high magnification.

In the course of the evolution of implant dentistry, advances in the form of implant surface modifications have led to stronger bone responses and higher implant survival rates [86]. Associations have been reported between significantly greater crestal bone loss and different implant surfaces and topographic features [86]. Furthermore, it has been suggested that surface roughness might have some role in the incidence of peri-implantitis [87] (Figure 11). In contrast, other authors consider that there are no available data confirming an association between implant surface features and the initiation of peri-implantitis or the progression of established peri-implantitis [88]. Ligature animal models have shown an increased risk of peri-implantitis with SLA implants in comparison to machined implants [89], and with TiUnite implants in comparison to machined, SLA, and TiOblast implants [31,90,91]. Similarly, another preclinical study noted significantly greater intrasurgical defect depths, defect widths, probing depths, and radiographic bone loss with TiUnite implants than with Straumann SLA or Biomet T3 implants [92]. Other factors apart from surface features might be relevant in the initial phase; for example, the invaginating grooves and pits on the TiUnite surface might favor bacterial adhesion and protect bacteria from shear forces [92]. Interestingly, a recent systematic review failed to find a long-term association between different surface modifications. Hence, these data in humans suggest that it is possible to achieve very good long-term results with all types of moderately rough implant surfaces [93].

Furthermore, the management of established peri-implantitis is possible after surgical treatment, but the therapeutic outcome is also influenced by the implant surface characteristics [90]. In a randomized clinical trial on the effect of adjunctive systemic and local antimicrobial therapy in patients with peri-implantitis, treatment success was reported in 79% of the implants with nonmodified surface features, but in only 34% of the implants with modified surfaces [94]. Similarly, a three-year randomized controlled trial found the surgical treatment of peri-implantitis to be effective, with stable peri-implant marginal bone levels, but here again the nonmodified surfaces yielded significantly better results [95].

Depending on the surface modification methods used, some remnants may persist on the surface and have deleterious effects upon the clinical performance of the implant [87]. These particles and others released from the surface as a result of corrosion and mechanical wear may have cytotoxic effects and stimulate inflammatory reactions [8], leading to osteoclast activation and further peri-implant bone loss. Likewise, it has been recently evidenced that titanium particles derived from implants containing phosphate-enriched titanium oxide, fluoride-modified, and grit-blasted (GB) surface treatments are able to activate CHK2 and trigger the recruitment of BRCA1 in oral epithelial cells. These are markers for detecting activation of the DNA damage response. Accordingly, it can be inferred that titanium particles released into a surgical wound may contribute to the disruption of epithelial homeostasis, and potentially compromise the oral epithelial barrier [96].

## 8. Other Perspectives to Conceive Peri-Implantitis

Different perspectives to understand peri-implantitis have been further proposed. As such, it has been advocated that peri-implant marginal bone loss might be related to a change in the foreign body equilibrium between the host immune system and the implant device [97]. The biological behavior of the bone cells is mediated by the interaction among immune cells (neutrophils, macrophages, and lymphocytes) and the dental implant (or any kind of foreign body). Briefly, one of the very first interplays around the foreign body is carried out by neutrophils for 24–48 h [98]. The insertion of a dental implant induces a status of hypoxia in the surrounding bone that leads to neutrophil accumulation in order to promote angiogenesis. Also, neutrophil cells may discharge proteolytic enzymes and reactive oxygen species during their function that can erode the implant surface and release metal particles to the tissues [99]. After 48 h, the population of macrophages is higher around the foreign body and these cells may lead the evolution of the immune response. In fact, these cells promote osteoclastogenesis, matrix deposition, and bone anabolism [100], whereas macrophages’ absence might impair osteoblast viability and bone formation [101,102]. Neutrophil apoptosis is mediated by macrophages during the shift between inflammatory phase to the healing phase. Also, the polarization between macrophages M1 to M2 and the length of each phase may have clinical effects, thereby an extended M1 phase may lead to a fibrous encapsulation of the fixture and implant failure [103]. On the contrary, higher presence of M2 macrophages has been reported on commercial pure implants [99,104], leading to bone deposition on the surface to isolate the fixture from the surrounding bone.

In addition, macrophages can differentiate into osteoclasts during bone remodeling and have phagocytosis capabilities until 5 μm of particle size [98]. Under presence of larger particles, macrophages tend to fuse to become foreign body giant cells (FBGCs). FBGCs are more frequent around the implant interface [105] than around healthy tissues and this fact might indicate the presence of a foreign body reaction around the dental fixture or the allogenic material.

Hence, under this concept of foreign body reaction, osseointegration is a mild chronic inflammatory and immunologically driven response where the bone–implant interface remains in a state of equilibrium but susceptible to changes in the environment [7,106]. Macrophages, FBGCs, and others approach this new bone barrier and if any disturbing factor occurs, a reactivation of the immuno-inflammatory cells against the foreign body material takes place. The loss of the foreign body equilibrium may thus stand as one of the reasons for this peri-implant bone loss [98].

## 9. Conclusions

Site-specific diseases are often attributable to local predisposing factors. In the case of plaque-associated peri-implantitis, local contributors, including surgical and prosthetic factors, as well as soft and hard tissue characteristics, may be predisposing factors to biofilm adherence around dental implants, thus leading to inflammation. Moreover, two major precipitant or triggering factors can be identified: residual cement and residual floss. In addition, current evidence seems to suggest that certain surface topographies can further accelerate the process of peri-implantitis.

## Figures and Tables

**Table 1:** Clinical characteristics of progressive peri-implant bone loss (peri-implantitis) based on the clinical findings.

Study	Study Design	Follow-Up (Mean)	Methods	Clinical Characteristics of Peri-Implantitis
**Fransson et al. 2005, 2008 [37,38]**	Cross-sectional	9.4 years	82 patients (197 implants with progressive bone loss/285 implants with stable bone)	BOP, SUP, recession, and PPD ≥ 6 mm were greater at implants with than without ‘progressive’ bone loss The proportion of affected implants that exhibited pus and PPD ≥ 6 mm was higher in smokers than in nonsmokers SUP, recession, and PPD ≥ 6 mm at an implant in a smoking subject had a 69% accuracy in identifying progressive bone loss
**Schwarz et al. 2017 [39]**	Cross-sectional	-	60 patients (36 healthy implants/26 with mucositis/167 with peri-implantitis)	Median PPD was 3 mm at healthy implant sites Median PPD was 4 mm at peri-implant mucositis sites Median PPD was 5 mm at peri-implantitis sites PPD (i.e., by tactile sensation) revealed that 135 of 167 implant sites were associated with a missing buccal bone plate
**Schwarz et al. 2017 [40]**	Cross-sectional	2.2 years	238 patients (216 implants with mucositis/46 implants diagnosed with peri-implantitis)	At mucositis sites, the BOP scores ranged between 33% and 50%, while the peak at peri-implantitis sites was 67% Diseased implant sites were associated with higher frequencies of 4–6 mm PPD versus healthy sites PPD values of ≥7 mm were only observed in one implant diagnosed with peri-implantitis
**Monje et al. 2018 [35]**	Case-control	3.17 years	141 patients (90 healthy implants/76 mucositis implants/96 peri-implantitis implants)	Sites with peri-implant mucositis showed significant levels of BOP (OR = 3.56), redness (OR = 7.66), and PPD (OR = 1.48) compared to healthy sites Sites exhibiting peri-implantitis showed significant levels of BOP (OR = 2.32), redness (OR = 7.21), PD (OR = 2.43), and SUP (OR = 6.81) compared to healthy sites PPD was the only diagnostic marker displaying significance comparing peri-implant mucositis and peri-implantitis sites (OR = 1.76) Tissue-level compared to bone-level implants were less associated with positive SUP (OR = 0.20) and PI (OR = 0.36)
**Ramanauskaite et al. 2018 [36]**	Cross-sectional	-	269 implants (77 healthy/77 mucositis/115 peri-implantitis)	In patients diagnosed with peri-implant mucositis, the mean BOP values amounted to 20.83% (43% at implant level) In patients diagnosed with peri-implantitis, the mean BOP values amounted to 71.33% (86% at implant level) In patients diagnosed with peri-implantitis, the mean SUP values amounted to 30.16% (17.39% at implant level) The mean PPD values at implant level were found to be 2.95 mm at healthy implant sites The mean PPD values at implant level were found to be 3.10 mm at peri-implant mucositis The mean PPD values at implant level were found to be 4.91 mm at peri-implantitis sites

BOP: bleeding on probing; SUP: suppuration; PPD: probing pocket depth; OR: odds ratio

**Table 2:** Conflicting findings concerning the significance of the presence or lack of keratinized mucosa around dental implants.

Study	Study Design	Implant Function Time (Mean)	Methods	Clinical, Radiographic, and Patient-Reported Outcomes
**Wennstrom et al. 1994 [46]**	Prospective	5–10 years	39 patients (171 Branemark pure titanium implants)	24% of the implants presented with no KM 13% of the implants presented with <2 mm of KM 39% of the implants had attached mucosa Neither attached nor keratinized mucosa were associated to peri-implant conditions
**Romanos et al. 2015 [50]**	Cross-sectional	9.4 years	118 patients (320 implants) Platform switched dental implants	A wide band of ≥2 mm of KM was associated with a significantly lower mBI (0.12 ± 0.37; *p* < 0.0001), plaque index (0.45 ± 0.56; *p* = 0.001), and less mucosal recession (0.06 ± 0.23; *p* < 0.0001) than a narrow band of KM (<2 mm) Considering regular and irregular implant maintenance therapy, a statistically significant difference was found between wide and narrow width of KM In irregular compliers, the presence of KM is a protective mechanism for better peri-implant conditions
**Roccuzzo et al. 2016 [51]**	Prospective	10 years	98 patients	The absence of KM was associated with greater plaque accumulation, greater soft-tissue recession, and a larger number of sites requiring additional surgical and/or antibiotic treatment Patient-reported outcomes regarding maintenance procedures showed major differences between the groups, favoring the presence of KM
**Bonino et al. 2018 [52]**	Prospective	6 months	238 patients (216 implants with mucositis/46 implants diagnosed with peri-implantitis)	Patients without peri-implant KM were less satisfied with the esthetics of the soft tissues around their implants (*p* < 0.01) Lack of KM was not associated with discomfort during brushing There was greater recession around implants without KM after 3 months (*p* < 0.01), but not after 6 months
**Perussolo et al. 2018 [53]**	Prospective	4 years	54 patients (202 implants)	The values of the clinical parameters were greater in the <2 mm KM band: mean mPI (0.91 ± 0.60), BOP (0.67 ± 0.21), and BD (12.28 ± 17.59) Marginal bone loss was greater in the KM < 2 mm group (0.26 ± 0.71) than in the KM ≥ 2 mm group (0.06 ± 0.48) KM width and time in function had a statistically significant effect on marginal bone loss In the <2 mm KM group, 51.4% presented with discomfort during brushing
**Monje et al. 2018 [49]**	Cross-sectional	5.73 years	37 patients (66 implants: 26 implants <2 mm/40 implants ≥2 mm) Erratic maintenance compliers (<2×/year)	On comparing a KM band of <2 mm versus ≥2 mm, and with the exception of suppuration (*p* = 0.6), all the clinical and radiographic parameters were significantly increased when the KM band was <2 mm (*p* < 0.001) A significant correlation was observed between KM and KT (r = 0.55) A lack of KM did not condition a lack of KT In the presence of peri-implantitis, only bleeding on probing at the adjacent dentate sites was seen to be increased Patients had no brushing discomfort with a mean band of KM ≥ 2.5 mm

KM: keratinized mucosa; KT: keratinized gingival tissue; mBI: modified bleeding index; BOP: bleeding on probing; mPI: modified plaque index; BD: brushing discomfort.
